# Characterization of K-Complexes and Slow Wave Activity in a Neural Mass Model

**DOI:** 10.1371/journal.pcbi.1003923

**Published:** 2014-11-13

**Authors:** Arne Weigenand, Michael Schellenberger Costa, Hong-Viet Victor Ngo, Jens Christian Claussen, Thomas Martinetz

**Affiliations:** 1 Institute for Neuro- and Bioinformatics, University of Lübeck, Lübeck, Germany; 2 Graduate School for Computing in Medicine and Life Science, University of Lübeck, Lübeck, Germany; 3 Institute for Medical Psychology and Behavioral Neurobiology, University of Tübingen, Tübingen, Germany; 4 Computational Systems Biology, Jacobs University Bremen, Bremen, Germany; Oxford University, United Kingdom

## Abstract

NREM sleep is characterized by two hallmarks, namely K-complexes (KCs) during sleep stage N2 and cortical slow oscillations (SOs) during sleep stage N3. While the underlying dynamics on the neuronal level is well known and can be easily measured, the resulting behavior on the macroscopic population level remains unclear. On the basis of an extended neural mass model of the cortex, we suggest a new interpretation of the mechanisms responsible for the generation of KCs and SOs. As the cortex transitions from wake to deep sleep, in our model it approaches an oscillatory regime via a Hopf bifurcation. Importantly, there is a canard phenomenon arising from a homoclinic bifurcation, whose orbit determines the shape of large amplitude SOs. A KC corresponds to a single excursion along the homoclinic orbit, while SOs are noise-driven oscillations around a stable focus. The model generates both time series and spectra that strikingly resemble real electroencephalogram data and points out possible differences between the different stages of natural sleep.

## Author Summary

In recent years, sleep has drawn increasing attention due to its multifunctional role, e.g. the involvement in the consolidation of memory. While neural mass models have been successfully employed to describe the dynamics of the awake brain, the drastic changes that arise during sleep have been challenging. As intracellular recordings point to a bistability in the membrane voltage of individual neurons, previous studies assumed a bistability to be responsible for the generation of SOs as well as KCs on the macroscopic scale. Here we present a minimal neural mass model of the cortex that we extend by a slow firing rate adaptation, which is assumed to underlie the termination of the cortical up state. A bifurcation analysis reveals the existence of a Hopf bifurcation together with an canard phenomenon. We show that these additional bifurcations are able to generate KCs as well as SOs, and reproduce the electroencephalogram (EEG) of sleeps stages N2 and N3 to a high degree. Based on these findings, we propose a new route for the sleep/wake transition, that is also consistent with the effect of neuromodulators on the brain.

## Introduction

Several studies indicate a major role of slow wave sleep (SWS) in the consolidation of memories [1], [2]. Especially its hallmarks, cortical slow oscillations (SO), are hypothesized to be a key mechanism for the transfer of memory into the neocortical long-term storage [3], [4]. Furthermore, it has been shown that the efficacy of memory consolidation can be improved with oscillatory transcranial electric and phase-locked auditory stimulation [5]–[7].

In the human electroencephalogram (EEG) SOs are defined as waves with a frequency of 0.5–2 Hz and a peak-to-peak amplitude >75 *µ*V [8], [9]. Underlying the SO is a widespread, almost synchronous alternation of neocortical networks between phases of depolarization (active or up state) and hyperpolarization (silent or down state) [10], [11], that behaves like a traveling wave [9], [12]. Modeling and experimental studies indicate a role for both, synaptic mechanisms and intrinsic currents, in the generation of SOs [13]–[16].

The K-complex (KC) occurs at the pace of the SO [17] and is believed to be the EEG expression of the cellular slow oscillatory activity [18]. The negative peak of the KC marks the transition to the cellular up state [19]. A KC during light NREM sleep (N2) was identified to be an isolated down state [20]. Furthermore, KCs show a high variability in morphology and amplitude, but are generally characterized as a negativepositive event with a sharp negative peak. Common variations of this theme are multiple peaks in the negative component or an initial positive bump before the negative-positive complex.

The components of evoked KCs were found to have typical latencies, namely the P200, N550 and P900 peaks. It was suggested that these components are not independent and share a common generation mechanism. Sometimes later components (N1500, P1900) with smaller amplitude are reported too [21], [22].

The complexity of the brain on the structural as well as the neuronal level has, however, been challenging for theoretical studies and modeling approaches. Neural mass models, pioneered by the work of [23] and [24], successfully described many phenomena of the human EEG, e.g. alpha and gamma rhythms, evoked responses and epilepsy [25]–[27]. See [28] and [29] for reviews.

In addition to states of wakefulness sleep has been modeled within the neural mass framework, too. A parameter study by [30] revealed the importance of synaptic gains for the dominant frequency of neural mass models. Steyn-Ross et al. [31] investigated the effect of changes in the efficacy of excitatory connections and the resting membrane voltage, finding multiple stable states which they classified as sleep and wake.

While those features are generated within a -local- column of neural tissue, spatial components have been shown to lead to complex interactions with the intrinsic dynamics, e.g. Turing patterns and traveling waves [32], [33]. However, within this study we focus on the generation of KCs as well as SOs rather than their spatial propagation. Nevertheless, our model can form the basis of a network of neural masses that covers spatial aspects, such as wave propagation.

Activity-dependent feedback via slow potassium channels has been suggested as a mechanism for the generation of SOs and KCs because of their sensitivity to the sleep related neuromodulator acetylcholine and their implication in the slow afterhyperpolarization [34], [35]. Multiple studies also point out that potassium leak channels can be activated by several anesthetics [36]–[38]. In the neural mass framework additive and multiplicative adaptation mechanisms have been discussed by [39]–[41]. So far KCs were described as excursions from a stable silent state to an unstable active state and the related SOs as oscillations between those two states [42], [43].

However, while for certain forms of anesthesia it seems plausible that the cortex undergoes a phase transition, it is not clear whether this necessarily holds for natural sleep [39], [44]. Addressing these issues we present a neural mass model for the sleeping cortex which is extended by sodium dependent potassium current [45], [46]. This approach links our neural mass model to modeling studies on SO generation based on single neurons as well as to experimental studies [14], [16]. The model output resembles EEG time series of sleep stages N2 and N3 to a high degree and shows key features of spontaneous and evoked KCs.

Building upon a bifurcation analysis, we characterize the dynamic repertoire of the cortex model. Our analysis indicates that cortical SOs and KCs are related but different phenomena. We suggest a route for the transition from wake to deep sleep and point out differences between natural sleep and anesthesia.

## Methods

In the following sections, we motivate and describe the mathematical foundation of the model. First, we introduce the concept of neural mass models and define the basic neural module we use as a starting point. Into this basic neural module, we incorporate a physiological plausible firing rate adaptation, characteristic for the sleeping cortex.

### Neural mass framework

Instead of considering single neurons individually, an averaged representation of the respective neuron type describes the behavior of a whole population. The mean membrane voltage 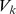 of the neural population 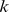 is transformed into a firing rate 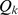 via a sigmoidal mapping [25], [47].
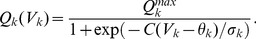
(1)


Here, 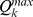 is the maximal firing rate of the respective population, while 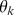 represents the firing threshold of the population and 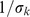 the gain or steepness of the transition. 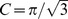 acts as a scaling factor that links the gain directly to the standard deviation of the change in firing rate 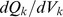.

At the dendrites, incoming spikes elicit transmitter release leading to the opening of synaptic channels. At any time, the fraction of open channels 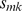 of type 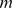 at population 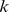 can be described by a convolution with an alpha function 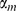 [48], with
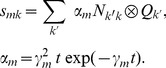
(2)


Here, the inverse rise time 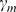 determines its shape. The sum is over all spikes from different sources 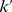 that arrive at the same type 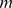 of synapses at population 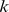. We consider AMPAergic synapses for excitation and a generic GABAergic type for inhibition, leading to the second order differential equations
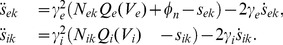
(3)


Here, 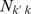 stands for the mean number of synaptic connections of type 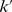 to population *k*. While inhibitory populations only spread locally, there are two different sources of excitation: local inputs 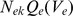 and background noise coming from unspecified brain structures 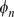, which is taken as uncorrelated Gaussian white noise with zero mean. To model external stimulation the mean of the background noise is elevated by 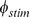 representing increased incoming spike rates.

**Figure 1:**
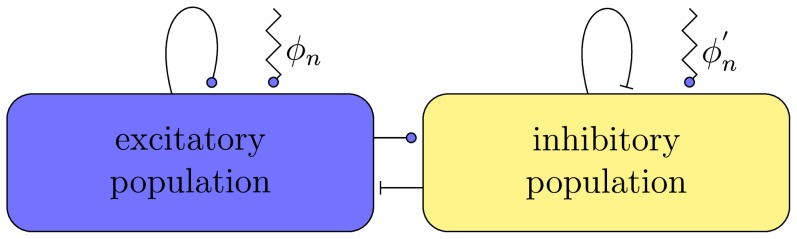
Connectivity of the cortex. The two populations are all to all coupled. In addition to intrinsic activity both populations receive background noise from unspecified brain structures. Circles indicate excitatory synapses, butts inhibitory synapses.

The connectivity structure of our model is given in Figure 1. It consists of an excitatory and an inhibitory population coupled all to all.

An important assumption of most neural mass models is the existence of an equilibrium state 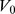 the system is always close to [23]. However, this is not true for KCs and SOs and the scaling of synaptic currents with respect to the membrane voltage 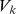 becomes important. This was addressed by [49] with the introduction of a weighting term 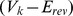.

Their model can be written similar to the classical conductance based form of [50] with one leak and two synaptic currents as
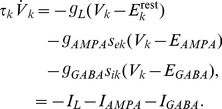
(4)


Here, 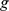 depicts the maximal conductivity, and 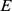 corresponds to the Nernst potentials of the respective channel.

The potential fluctuations measured in an EEG are mainly generated by pyramidal neurons [51]. Therefore, we use the membrane voltage of the excitatory population as our output variable. Similarly, multiple studies [25], [49], [52]–[54] used either the deviation of the membrane voltage from the resting state, 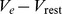, or the membrane voltage itself. As our system has no spatial extension and we only assume ohmic effects of skull and scalp, the EEG signal can be approximated by a linear scaling of the excitatory membrane voltage. When comparing experimental data and model output both time series are z-scored, because this linear transformation normalizes mean and standard deviation but preserves the other statistical properties of a signal. As we are only interested in qualitative properties of the model, e.g. the ratio between medium amplitude background oscillations and large amplitude deflections during N2, the different sleep stages are z-scored independently. For quantitative statements the same measuring function must be used.

### Model extension with respect to sleep

As motivated in the introduction, we add the sodium dependent potassium current
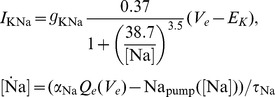
(5)to the excitatory population, see Equation 4. The current is connected to the excitatory membrane voltage by a membrane capacity *C_m_* = 1* µ*F/cm. Sodium influx responsible for 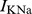 activation results from spiking or 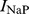 activation, for which a depolarization above −60 mV is sufficient. We do not explicitly model these mechanisms but combine their effects via the *V_e_*-determined spike rate 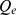 and regard 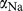 as average sodium influx per spike. Sodium extrusion is due to an active pump [55], which is detailed in Text S1. For simplicity, we neglect synaptic depression and other candidate mechanisms for additive feedback, like calcium dependent potassium currents.

This approach is qualitatively different to changes in the firing rate function, as utilized by [39]. Gradually switching between two firing rates alters the overall shape of the sigmoid function in a multiplicative activity-dependent manner, whereas we employ an additive threshold modulation.

### Computational methods

The model was implemented in C++ and run within MATLAB [56]. The stochastic differential equations were iterated using a stochastic Runge-Kutta method of 4th order [57] with a step size of 0.1 ms. Simulation length was chosen as 30 s with a 5 s onset to ensure a steady state. All settings were run multiple times to check for robustness. Full model equations and parameters are given in Text S1 and Table S1. Bifurcation analysis is done with XPPaut [58], and a script is provided in Text S2.

## Results

In the following, we analyze the dynamic repertoire of the model and define multiple dynamic regimes. We stress that the bifurcation analysis is done in the *noise free deterministic* case.

However, noise is able to push the system into different regimes, if the parameters are chosen sufficiently close to the border. Therefore, the analysis provides the repertoire of *possible* modes, whereas the corresponding response to external stimuli is captured in a following section.

Based on that description we show that the model is able to reproduce KCs as well as SOs. Furthermore, the analysis suggests a distinction between KCs and SOs and provides some insights in the differences between natural sleep and anesthesia. We will finally present a comparison of the noisy simulation to human EEG data.

### Bifurcation analysis

In order to characterize the dynamic repertoire of the cortical model we conducted a numerical bifurcation analysis of the noise-free system. The qualitative behavior of the model was most sensitive to changes in the inverse gain, 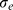, of the pyramidal population and the strength of the adaption, 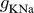.

Additionally, both parameters are known to be susceptible to changes in the neuromodulatory milieu, and the concentration of many major neuromodulators is known to change throughout the sleep-wake cycle. Cortical acetylcholine levels are lowest during slow wave sleep and highest during wake and REM sleep, whereas serotonin and norepinephrine levels are highest during wake, intermediate during SWS and lowest during REM sleep [59].

Tonic application of acetylcholine blocks leak and activity-dependent potassium currents 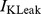, 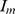, 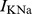, 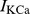 (reviewed in [60]), as well as 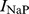 [61]. Furthermore, many studies show that 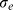 can be altered by norepinephrine, serotonin, acetylcholine as well as dopamine [35], [62]–[69].

Consequently, 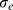 and 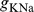 were chosen as bifurcation parameters. The adaptation currents are primarily found in excitatory pyramidal cells and less so in inhibitory interneurons, which justifies the restriction of the parameter changes to the excitatory population.

**Figure 2:**
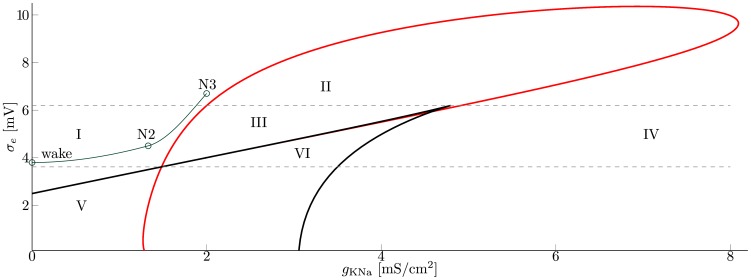
Bifurcation diagram of the cortex with respect to 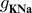 and 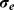. Overview over the models dynamic regimes, obtained via numerical bifurcation analysis of the cortex with respect to 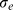 and 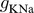. Hopf bifurcations are drawn in red, while the black line depicts saddle-node bifurcations. The bottom gray line marks the intersection of Hopf and saddle curves, the top gray line the cusp bifurcation. The green line depicts the proposed route for the transition from wake to sleep stage N3. The region around wake corresponds to parameter settings commonly used for wake EEG. N2 and N3 are settings used within this study for the respective sleep stages, as given in Table 2 and 3. Regions I-VI are described in the text and Table 1 (Parameters as in Table S1).

**Figure 3:**
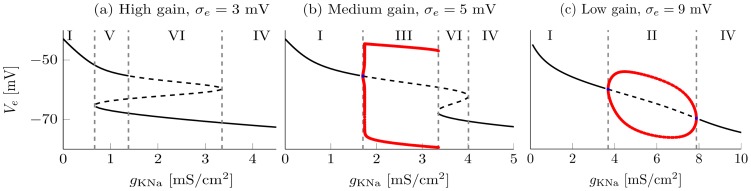
One-dimensional bifurcation diagrams for different gain levels 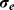. Low gain corresponds to high values of 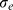. Thick black lines depict stable fixed points, dashed lines unstable fixed points and red lines stable periodic solutions. The gray dashed lines mark bifurcations and separate the different regimes. (a) Two saddle-node bifurcations lead to excitability in region VI and bistability in region V. (b) A Hopf bifurcation appears (between I and III) in addition to the two saddle-nodes. The initial small amplitude limit cycle transitions into a high-amplitude relaxation cycle via a canard explosion. The high-amplitude periodic solutions vanish at the left saddle-node via a homoclinic bifurcation. The period of the relaxation oscillations goes to infinity as one approaches the homoclinic bifurcation. (c) Only the Hopf bifurcation remains, after the saddle nodes disappeared via a cusp bifurcation. Within region II there is no canard anymore.

As can be seen in Figure 2 the dynamics of the system is shaped by two bifurcations. The first one is a fold created by two saddle node bifurcations (black), that vanishes in a cusp. Between the two saddle nodes there are three equilibrium states, leading to bistability or excitability, see Figure 3a or Figure 3b. This is in good agreement with [31] and [70], as in the case of a fixed sodium concentration 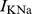 is constant, and an increase in 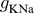 acts as a decrease in resting potential.

The second bifurcation is a Hopf arising at the upper stable branch (red). Importantly there is a canard explosion, where the small amplitude limit cycle of the Hopf bifurcation transitions into a high-amplitude relaxation cycle. This phenomenon was first described by [71] and is typical for systems where fast and slow subsystems interact. The relaxation cycle vanishes at the left saddle-node via a homoclinic bifurcation. At the cusp both saddle nodes coalesce and the homoclinic bifurcation turns into a second Hopf point.

Based on those bifurcations we define multiple dynamical regimes, see Table 1 for a short overview. Within region I a single stable state exists at depolarized membrane voltages where the cortex shows relatively high activity (see Figure 3). Especially for small values of 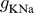 even large excitatory and inhibitory inputs only cause a passive response. A switch to the lower branch of the S-shaped curve in Figure 3 (region IV, silent state) is not possible. Because of these properties we assume the waking brain to operate within this regime.

**Table 1:** Dynamic regimes of the cortical module.

Region	dynamical properties
I	active cortex
II	limit cycles
III	limit cycles and relaxation cycles
IV	silent cortex
V	bistable
VI	excitable

When crossing the curve of saddles to region V two new fixed points appear (see also Figure 3a). The system becomes bistable, with a stable active and silent state. Positive and negative inputs can cause a switching between the two stable branches.

A further increase in 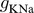 turns the upper branch (active state) unstable. However, within region VI there are still multiple equilibria leaving the system excitable. Here a stimulus can produce a large positive response, which was previously thought to be responsible for the generation of KCs as well as SOs [72].

Only after the second saddle node is crossed the upper two equilibria vanish and a single stable state remains. This state is characterized by hyperpolarized membrane voltages leading to a quiescent cortex.

Region III is characterized by periodic limit cycles or relaxation oscillations and, hence, high rhythmicity. The initial Hopf bifurcation is accompanied by a canard explosion: due to an exponentially small variation of the bifurcation parameter an abrupt transition from a medium-amplitude limit cycle to a high-amplitude relaxation cycle can take place.

This phenomenon was first described in [71] and is typical for systems where fast and slow subsystems interact. The corresponding one-dimensional bifurcation diagram is shown in Figure 3b. The periodic solutions vanish at the left saddle-node via a homoclinic bifurcation, and the period of the relaxation oscillations goes to infinity as one approaches the homoclinic bifurcation.

Additionally, with increasing 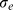 the amplitude of the limit cycle increases and approaches the form of relaxation oscillations. This explains the similarity between the limit cycles and relaxation oscillations. Both are shaped by the same homoclinic orbit.

At the cusp the two saddle nodes vanish and the homoclinic bifurcation turns into a second Hopf point. Without the homoclinic bifurcation there is no canard anymore. Therefore, in region II above the cusp bifurcation only limit cycles remain, illustrated in Figure 3c, leading to high-amplitude oscillations.

### Response to perturbations

While the bifurcation analysis provides the basic repertoire of the unperturbed model, its responsiveness with respect to perturbations, e.g. external stimuli or background noise, is crucial for its behavior. As mentioned before, within region I the cortex shows only a passive response. However, this changes for larger values of 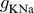, i.e. closer to the curve of Hopf points (red line in Figure 2, separating region I from II and III).

**Figure 4:**
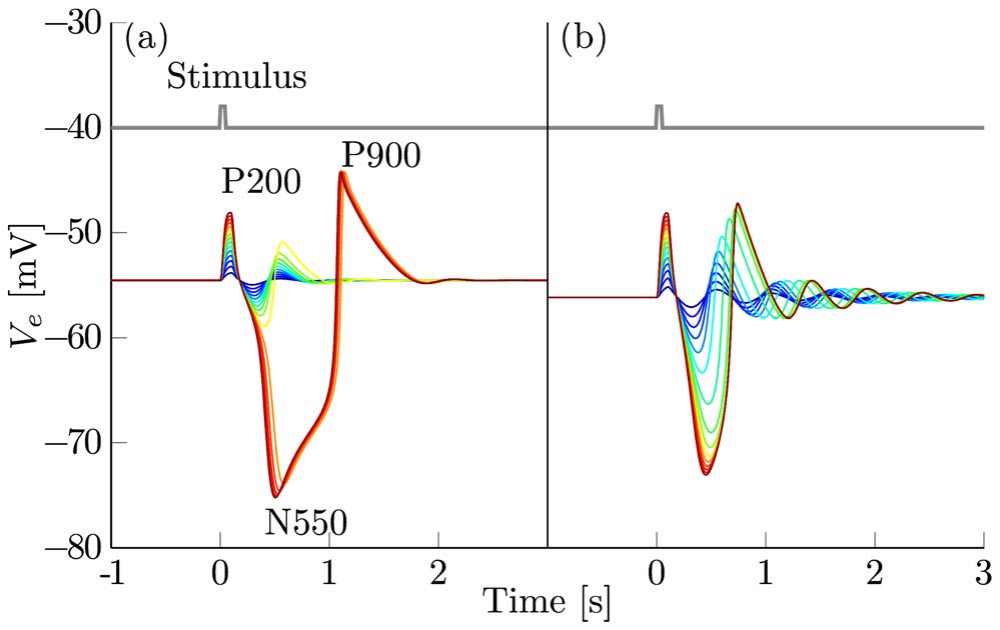
Response of the noise-free cortex to artificial stimuli. Excitatory bursts of 50 ms duration were applied to both populations. The spike rate of the stimuli 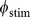 varies uniformly from 5 Hz (dark blue) to 100 Hz (dark red). The stimulus is shown in grey. (a) Bifurcation parameters are set to the mark N2 close to region III (see Table 2). There, a canard explosion leads to large amplitude responses that qualitatively resemble a typical evoked KC with its P200, N550 and P900 components. (b) Parameters are set to mark N3, so that the cortex is beyond the cusp close to region II (see Table 3). The canard vanished, leading to an even increase in the amplitude of the response.

There, positive as well as negative inputs may cause a reverse spike resembling a KC. Additionally, close to the curve of Hopf points the stable active state turns into a stable focus, i.e. the system behaves like a damped oscillator upon perturbation. In Figure 4a we show the response to artificial stimuli 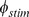 of varying strength, when the cortex is set close to the Hopf bifurcation between region I and III.

Stimuli of low strength lead to damped oscillations whose amplitudes are considerably larger than during the wake state but smaller than KCs or SOs. However, as the strength of the stimuli increases the system is pushed into the canard explosion and the amplitude of the response increases rapidly. While in Figure 4a there seems to be a threshold separating the two types of responses, it is actually a smooth transition given sufficiently small increases in stimulation strength.

The induced relaxation cycles show a good qualitative match with KCs seen during sleep. In the noise driven simulation the majority of inputs would lead to medium-amplitude oscillations, whereas only the rare outliers would trigger a KCs like response. This is in good agreement with the dynamics seen in sleep stage N2, where medium-amplitude background oscillations are interrupted by large amplitude KCs.

We assume this mechanism to be responsible for the generation of KCs during sleep stage N2. Furthermore, this requires the cortex to be in the *active* state close to the Hopf bifurcation to region III, rather than being in the silent down state. This is in good agreement with multiple studies who report that during SWS of naturally sleeping animals more time is spent in up states than in down states [73]–[78].

Close to the Hopf, an increase of the inverse gain, 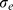, leads to an *increase* in the amplitude of the background oscillations and they approach the shape of a relaxation cycle. Beyond the cusp the canard vanishes and isolated events in the sense of KCs are not possible anymore (see Figure 4b).

This behavior is well reflected in what is seen during sleep stage N3, where SOs appear as large amplitude oscillations, that are *not* separated from the ongoing background activity. Furthermore, it explains the high similarity between KCs and SOs, as they are both shaped by the same homoclinic orbit. We hypothesize that during sleep stage N3 the cortex is in region I close to the Hopf bifurcation to region II.

Together these findings give rise to a new interpretation of the sleep/wake transition. At the transition to sleep stage N2, the cortex approaches the Hopf bifurcation close to region III, which shifts the EEG trace to higher amplitudes and lower frequencies compared to wake activity. By virtue of a canard explosion this background activity is then interrupted by single, isolated relaxation cycles. As sleep deepens further, the cortex follows the route depicted in Figure 2, while the amplitude of the background oscillations increases and ultimately approaches the form of a KC.

However, this is in contrast with the view that the cortex undergoes a phase transition when entering NREM sleep. Interestingly, a similar model was utilized to describe characteristics of anesthesia [39]. We can reproduce similar behavior, e.g. burst suppression in region VI (See Supplementary Figure S1).

### Reproduction of sleep stages N2 and N3

**Figure 5:**
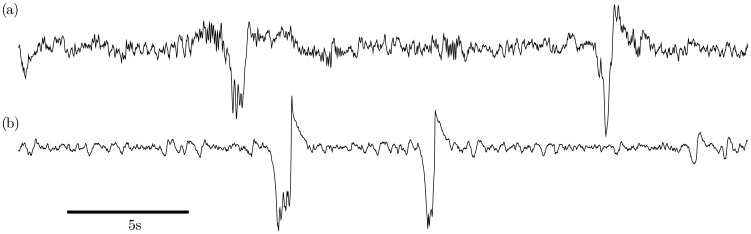
Comparison of human EEG with model output in regime N2. Qualitative comparison of (a) human EEG data of sleep stage N2 from electrode Cz with (b) the isolated cortical module in regime N2 (region IV in the bifurcation diagram in Figure 2). The traces illustrate the medium-amplitude background oscillations and the stereotypical shape of spontaneous KCs at the EEG level. It may or may not have an initial bump followed by a large negative peak and a pronounced positive overshoot. The model-KC is noise induced and represents a single relaxation cycle. An evoked KC in the noise-free case is shown in Figure 4a. Model output is excitatory membrane voltage *V_e_*, and both time series are z-scored (Parameters as in Table 2).

To verify the ability of the model to reproduce sleep stage N2 we set the model to parameter configuration “N2” from Figure 2 (See Table 2). The chosen parameter set is within region I close to the border of region III, an example time series is shown in Figure 5.

**Table 2:** Parameters of regime N2.

Symbol	Value	Unit	Description
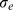	4.6	mV	inverse gain
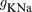	1.33	mS/cm^2^	conductivity

In a region close to the chosen parameters the cortex is in the up state and shows the expected noise-driven medium-amplitude oscillations. In addition, background noise may push the model into high-amplitude deflections that closely resemble KCs seen in human EEG. Similar to the data the KCs can show a single pronounced peak or a prolonged down state, which depends on the noise.

**Figure 6:**
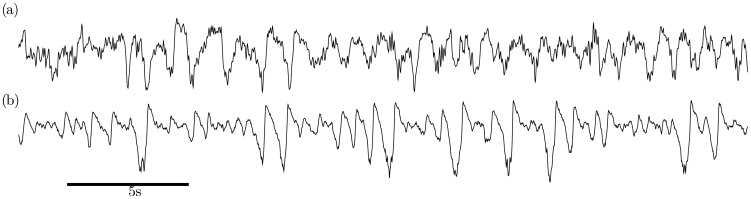
Comparison of human EEG with model output in regime N3. Qualitative comparison of (a) human EEG data of sleep stage N3 from electrode Cz with (b) the isolated cortical module in regime N3 (region I in the bifurcation diagram in Figure 2). As the system is close to the Hopf bifurcation noise leads to quasiperiodic oscillations around the stable focus (up state). Large amplitude oscillations resemble KCs as both are shaped by the same homoclinic orbit. The model output is excitatory membrane voltage *V_e_*, and both time series are z-scored (Parameters as in Table 3).

Following our route for the sleep/wake transition in Figure 2 we then moved along the Hopf bifurcation to a setting beyond the cusp and close to region II, labeled as “N3”. In Figure 6 a representative time series is shown with the parameters given in Table 3. There the cortex shows high amplitude oscillations around 0.8 Hz. In contrast to the N2 stage, the cortex does not produce KCs in the sense of isolated events that differ from the background oscillations. Rather, the response increases until it approaches the form of a KC, depending on the strength of the perturbation.

**Table 3:** Parameters of regime N3.

Symbol	Value	Unit	Description
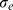	6.7	mV	inverse gain
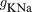	2	mS/cm^2^	conductivity

## Discussion

### Characterization of KCs and SOs

We explored an extended neural mass model of the cortex and related its multiple dynamical regimes to different sleep stages.

A bifurcation analysis revealed the existence of a fold as well as a Hopf bifurcation accompanied by a canard phenomenon. We argue that deflections generated by the canard explosion are identical to KCs seen in the EEG during natural sleep, leading to the spike-like nature of the KCs. Increasing the bifurcation parameter 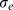 the canard vanishes, explaining the damped oscillatory behavior of SOs. Our analysis provides a clear theoretical distinction between KCs and SOs. However, as both the limit and the relaxation cycle are shaped by the same underlying homoclinic orbit, the actual transition is rather smooth even in the noise-free deterministic system (see Figure 4). Therefore, it might be challenging to find this distinction within experimental data.

Based on the bifurcation analysis we identified parameter regimes that show characteristics of sleep stage N2 and N3 and showed that our model is able to reproduce the EEG of both sleep stages to a high degree. Building upon these findings we propose an alternative scenario for the sleep wake transition. Rather than entering a bistable regime the cortex stays primarily within the active state. As sleep deepens, the cortex approaches the Hopf bifurcation, leading to an increase in amplitude and slowing of noise-driven background oscillations, as well as large amplitude deflections, i.e. KCs. At the transition to sleep stage N3 the canard phenomenon vanishes due to the cusp bifurcation. The remaining Hopf bifurcation is responsible for the generation of noise-driven SOs. Isolated events as in sleep stage N2 are not possible within that regime.

Parameter settings within region II or III lead to highly regular relaxation oscillations or limit cycles, that do *not* resemble human EEG. It is crucial that the cortex must be within region I *close* to region II or III to reproduce the data. In a study on resting state networks [79] found the awake brain to be in a state of criticality, which leads to an increased responsiveness. In this study, we also find the sleeping cortex close to a phase transition and suggest that the concept of criticality is not restricted to wakefulness, but carries over to sleep. However, the phase transition and computational goal are different.

Due to the presence of noise bifurcations do not lead to clear-cut qualitative changes of the dynamics [43]. Noise can shift critical points or induce behavior that is not seen in the deterministic case, such as noise-induced transitions.

### Relation to intracellular recordings

Our work deals primarily with the characteristics of EEG signals during NREM sleep. However, the presented bifurcation analysis is useful in a broader context. Similar activity is found e.g. during non-REM sleep, anesthesia, coma and in isolated cortical preparations. It becomes increasingly clear that there exists a continuum of slow oscillatory states, which are mainly characterized by the fraction of time spent in up or down states, the temporal regularity with which state transitions occur and the response to external stimuli.

The phenomenon of up and down states in intracellular recordings is commonly associated with the notion of bistability or relaxation oscillations. However, it is important to note that most results on SOs were obtained in deeply anesthetized animals or slice preparations. Under these conditions, the system is down state dominated, i.e. down states last longer than up states, the occurrence of up states is often highly rhythmic [76], [80] or up states are infrequent and transient [81]. In our model these classical regimes are also present, namely in regions III, V and VI.

Generally, SOs produced by anesthesia are much more regular than during natural sleep [76], [82]. Under ketamine-xylazine anesthesia neurons spend twice the time in silent states compared to natural SWS [76], and in the auditory cortex of awake rats prolonged up states are not even observed at all [83]. Furthermore, SO properties differ from one anesthetic to the other [84]. Ketamine-xylazine anesthesia produces a uniform and continuous SO state [85], whereas with urethane epochs of stable SOs are short-lived and desynchronized periods may occur spontaneously [86]. This is similar to SWS where one finds waxing and waning of slow wave complexes interleaved with periods reminiscent of active states [74].

In contrast, [20] pointed out that a KC during light sleep is not always embedded in an ongoing SO, but is mostly an isolated event. Clearly, in N2 the active state dominates. Similarly, many studies report that during SWS of naturally sleeping animals more time is spent in up states than in down states [73]–[78] Furthermore, it has been reported that SWS contains many episodes of low-amplitude fast oscillations, lasting several seconds and resembling the active state [87]. This evidence points to natural sleep being up state dominated.

Furthermore, bistability is inferred via bimodality in the distribution of individual cells' membrane potential. In local field potentials, one can observe a markedly conserved waveform of individual SO events [88], but bimodality is already less visible. It is known that collective dynamics can exhibit, e.g. limit-cycle regimes, but at the same time emerge from irregular and high-dimensional neuronal activity, which is only apparent at small-scales [89].

The spectrum of SO phenomena cannot be fully captured by the concepts of bimodality or relaxation oscillations. Our analysis corroborates that the KC can be identified with a single, isolated relaxation cycle and slow wave activity, including prolonged episodes of low-amplitude fast oscillations, stems from noise-driven oscillations around a stable focus. Down states occur frequently in the up state dominated cortex, but they are transient.

### Predictions

The assumption that a substantial gain change accompanies the change of sleep stages is reasonable, but still has to be clearly demonstrated experimentally for natural sleep. The only publication we are aware of that touches this issue is [73]. Our model indicates that an increase in gain can induce a bistable state when awake, moving from region I to region V. Likewise, looking at comatose states (region IV) a decrease in gain should induce limit cycle oscillations.

Additionally, constant neural activation, i.e. arousal, causes relaxation oscillations in the model. Indeed, this phenomenon seems to occur in comatose patients, too, where one observes an increase in delta activity after stimulation [90]. This is termed paradoxical arousal and should not be confused with the paradoxical excitation/biphasic response during the induction process of anesthesia.

Furthermore, given the suggested role of gain change in the transition between N2 and N3, an altered slope of the f-I-relation of excitatory pyramidal cells could be a key factor in distinguishing wake and REM sleep.

Activity-dependent and leak potassium currents (or tonically activated extrasynaptic 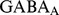 receptors) are both able to promote bistability in a cortical population. However, only activity-dependent mechanisms contribute to rhythmicity. It would be interesting to see their contributions revealed for natural sleep and anesthesia.

A study by Molaee-Ardekani et al. [39] showed that a similar model of slow firing rate adaptation can reproduce the effects seen under anesthesia. A comparison of our findings with their results suggest that the region of bistability (V) as well as the region of excitability (VI) are actually associated with anesthesia.

### Sleep: More than bistability and relaxation oscillations

A main result of this paper is that on the macroscopic level the cortex is not necessarily in a bistable regime during natural deep sleep. We argue that properties of KCs and SOs at the EEG level support the view of a monostable active cortex close to a Hopf and a saddle node bifurcation.

We stress that the characterization of KCs and SOs is made on the *population* level. While the switching between up and down states on the cellular level points to relaxation oscillations or bistability with noise-driven transitions, relatively regular oscillation at the cellular level may appear less regular at the EEG level, due to varying spatial synchrony [82]. Relaxation oscillations in the EEG usually correspond to pathological conditions like epilepsy.

We have not explicitly analyzed other adaptation mechanisms like multiplicative feedback arising due to synaptic depression or depletion of extra-cellular calcium or inhibitory modulation [91]. However, the additive activity-dependent feedback investigated here is sufficient to account for a multitude of phenomena in healthy and pathological conditions. Furthermore, we expect that the bifurcation structure of the system, i.e. presence of saddle-nodes, Hopf and homoclinic bifurcation, will persist in alternative settings. Thus, our main conclusions do not depend on the particular choice of the feedback mechanism.

## Supporting Information

Figure S1 **Burst suppression in region VI.** Within region VI the system shows characteristics of burst suppression. A quiescent stable down state is interrupted by large amplitude excursions around the unstable active state.
(TIF)

Table S1 **Parameters.** Description and values of all parameters that are not subject of the bifurcation analysis.
(PDF)

Text S1 **Full model equations.** Full mathematical description of the model used within this study.
(PDF)

Text S2 **Bifurcation analysis code.** Script file used for the bifurcation analysis in XPPaut.
(PDF)

Video S1
**Visualization of the sleep-wake transition.** This video illustrates the change of the evoked response to perturbations, as the model follows the proposed sleep-wake transition depicted in Figure 2. Close to the “wake” state, the system immediately returns to the stable fixed point, without any oscillatory behavior. At the onset of sleep, 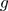 *K*Na and *σ*e increase and the system approaches the Hopf bifurcation, such that perturbations away from the active state lead to transient, small and slow oscillatory responses. However, as there are no large deflections, which resemble KCs this regime corresponds to sleep stage N1. Close to label “N2” in Figure 2, KCs emerge as isolated events through a canard explosion (Sleep stage N2). With further transition into deeper sleep, the amplitude of the background oscillations increases and approaches the relaxation cycle of the canard. Finally in the proximity of “N3” in Figure 2 there are no KCs in the sense of isolated events, but large amplitude slow oscillations around a stable focus (Sleep stage N3).(MP4)
