# Supplementary figures and images for: Characterization of K-Complexes and Slow Wave Activity in a Neural Mass Model
Source: PLoS Comput Biol. 2014 Nov 13;10(11):e1003923. doi: 10.1371/journal.pcbi.1003923 (PMC4230734; doi:10.1371/journal.pcbi.1003923)

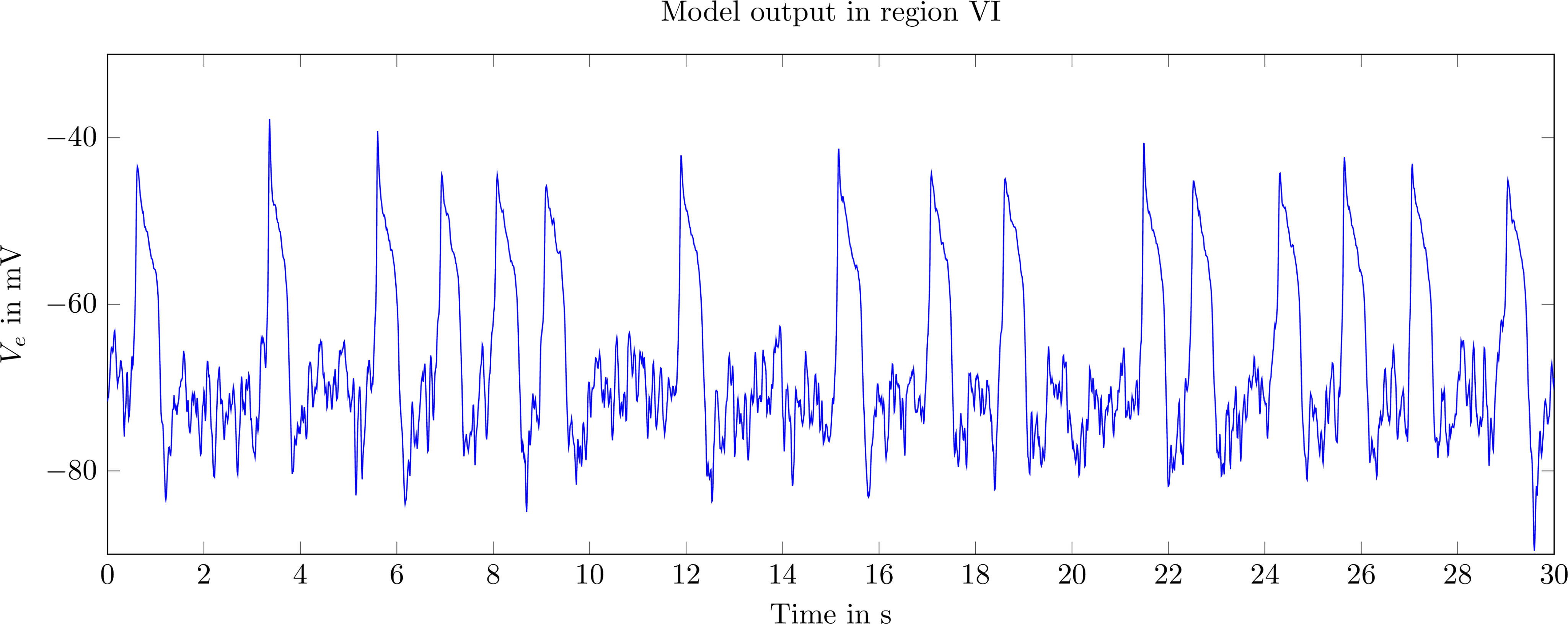

Supplement: Figure S1 — Burst suppression in region VI. Within region VI the system shows characteristics of burst suppression. A quiescent stable down state is interrupted by large amplitude excursions around the unstable active state. (TIF) [file pcbi.1003923.s001.tif]
