# Supplementary material for: Characterization of K-Complexes and Slow Wave Activity in a Neural Mass Model
Source: PLoS Comput Biol. 2014 Nov 13;10(11):e1003923. doi: 10.1371/journal.pcbi.1003923 (PMC4230734; doi:10.1371/journal.pcbi.1003923)
Supplement: Table S1 — Parameters. Description and values of all parameters that are not subject of the bifurcation analysis. (PDF) [file pcbi.1003923.s002.pdf]

Supporting Information Tables

Table S1 Parameter values

This table defines all constants used within the model, that are not described elsewhere.

Table 1. Parameter definitions

Symbol	Value	Unit	Description
τ_e, τ_i	30	ms	membrane rise time
Q_e^{max}	30E-3	ms^{-1}	maximal firing rate
Q_i^{max}	60E-3	ms^{-1}	maximal firing rate
θ_e, θ_i	-58.5	mV	firing threshold
σ_i	6	mV	firing rate deviation
γ_e	70E-3	ms^{-1}	synaptic rate constant
γ_i	58.6E-3	ms^{-1}	synaptic rate constant
N_{ee}	120	-	connectivity e-e
N_{ei}	72	-	connectivity e-i
N_{ie}	90	-	connectivity i-e
N_{ii}	90	-	connectivity i-i
C_m	1	$\mu\mathrm{F/cm}^2$	membrane capacity
g_L	1	$\mathrm{mS/cm}^2$	channel conductivity
E_{Le}, E_{Li}	-66,-64	mV	reversal potential
E_{K}	-100	mV	reversal potential
E_{AMPA}	0	mV	reversal potential
E_{GABA}	-70	mV	reversal potential
α_{Na}	2	mM/ mA ms	sodium influx
τ_{Na}	1	ms	sodium time constant
R_{pump}	0.09	mM ms^{-1}	sodium pump capacity
Na_{eq}	9.5	mM	sodium resting state
ϕ_n	120E-3	ms^-1	background noise standard deviation
