# Supplementary material for: Characterization of K-Complexes and Slow Wave Activity in a Neural Mass Model
Source: PLoS Comput Biol. 2014 Nov 13;10(11):e1003923. doi: 10.1371/journal.pcbi.1003923 (PMC4230734; doi:10.1371/journal.pcbi.1003923)
Supplement: Text S1 — Full model equations. Full mathematical description of the model used within this study. (PDF) [file pcbi.1003923.s003.pdf]

Supporting Information

Text S1 Model equations

The basic neural column described in Methods is given by the following set of equations:

$$\begin{aligned}
\tau_e \dot{V}_e &= -I_L - I_{AMPA}(s_{ee}) - I_{GABA}(s_{ie}) - \tau_e C_m^{-1} I_{\mathrm{KNA}}, \\
\tau_i \dot{V}_i &= -I_L - I_{AMPA}(s_{ei}) - I_{GABA}(s_{ii}), \\
\ddot{s}_{ee} &= \gamma_e^2 \left(N_{ee} Q_e(V_e) + \phi_n - s_{ee}\right) - 2\gamma_e \dot{s}_{ee}, \\
\ddot{s}_{ie} &= \gamma_i^2 \left(N_{ie} Q_i(V_i) \qquad - s_{ie}\right) - 2\gamma_i \dot{s}_{ie}, \\
\ddot{s}_{ei} &= \gamma_e^2 \left(N_{ei} Q_e(V_e) + \phi_n' - s_{ei}\right) - 2\gamma_e \dot{s}_{ei}, \\
\ddot{s}_{ii} &= \gamma_i^2 \left(N_{ii} Q_i(V_i) \qquad - s_{ii}\right) - 2\gamma_i \dot{s}_{ii}, \\
\dot{[\mathrm{Na}]} &= (\alpha_{\mathrm{Na}} Q_e(V_e) - \mathrm{Na}_{\mathrm{pump}}([\mathrm{Na}]))/\tau_{\mathrm{Na}}.
\end{aligned}$$

The currents are given by the following equations:

$$\begin{aligned}
I_L &= g_L(V_k - E_L), \\
I_{AMPA} &= g_{AMPA} s_{ek}(V_k - E_{AMPA}), \\
I_{GABA} &= g_{GABA} s_{ik}(V_k - E_{GABA}), \\
I_{\mathrm{KNa}} &= g_{\mathrm{KNa}} \frac{0.37}{1 + \left(\frac{38.7}{[\mathrm{Na}]}\right)^{3.5}} (V_e - E_K).
\end{aligned}$$

The sodium pump is described by:

$$\mathrm{Na}_{\mathrm{pump}}([\mathrm{Na}]) = R_{\mathrm{pump}} \left(\frac{[Na]^3}{[Na]^3 + 3375} - \frac{[Na]_{eq}^3}{[Na]_{eq}^3 + 3375} \right).$$
