# Supplementary material for: Characterization of K-Complexes and Slow Wave Activity in a Neural Mass Model
Source: PLoS Comput Biol. 2014 Nov 13;10(11):e1003923. doi: 10.1371/journal.pcbi.1003923 (PMC4230734; doi:10.1371/journal.pcbi.1003923)
Supplement: Text S2 — Bifurcation analysis code. Script file used for the bifurcation analysis in XPPaut. (PDF) [file pcbi.1003923.s004.pdf]

Supporting Information

Text S2 Bifurcation analysis

Numerical bifurcation analysis was performed with XPPauth. The model description of the isolated cortex is given by the following ODE file:

```
!Naeq=9.5 !alphaNa=2  !Rpump=0.09
!EKNa=-100 !Ee=0  !Ei=-70
!gammaE=70e-3 !gammaI=58e-3 !gL=1
!fRe=30e-3 !fRi=60e-3
!VRe=-58.5 !VRi=-58.5
!bRi=6*sqrt(3)/pi
number gee=120,gie=70,gei=90,gii=90
bRe=b*sqrt(3)/pi
Napump = Rpump * ( Na^3 / (Na^3 + 3375) - Naeq^3 / (Naeq^3 + 3375))
fatigue = 0.37 / (1 + (38.7 / Na)^3.5)
ifatigue = gfatigue * fatigue * (Ve - EKNa)
fe(x) =  fRe / (1 + exp(-(x - VRe) / bRe))
fi(x) =  fRi / (1 + exp(-(x - VRi) / bRi))
Ve' = (-gL * (Ve - EL) - sei * (Ve - Ei) - see * (Ve - Ee)) / C - ifatigue
Na' = (-Napump + alphaNa * fe(Ve)) / tfatigue
Vi' = (-gL * (Vi - EL) - sii * (Vi - Ei) - sie * (Vi - Ee)) / C
see' = uee
uee' = gammaE * gammaE * (gee * fe(Ve) - see) - 2 * gammaE * uee
sii' = uii
uii' = gammaI * gammaI * (gii * fi(Vi) - sii) - 2 * gammaI * uii
sei' = uei
uei' = gammaI * gammaI * (gei * fi(Vi) - sei) - 2 * gammaI * uei
sie' = uie
uie' = gammaE * gammaE * (gie * fe(Ve) - sie) - 2 * gammaE * uie
init Ve=EL,Vi=EL,Na=0
par C=30,tfatigue=1.3,gfatigue=1.33,b=4,EL=-64
@ maxstor=1000000,total=3000,dt=1,meth=8dp
@ nmax=300,ntst=50,epss=1e-7,epsl=1e-7,epsu=1e-7,parmin=0,parmax=10,
@ autoxmin=0,autoxmax=10,autoymin=-100,autoymax=0, ds=1e-3,dsmin=1e-5,dsmax=0.5
done
```
